# Monitoring Behaviorally Induced Biochemical Changes Using Fluorescence Lifetime Photometry

**DOI:** 10.3389/fnins.2019.00766

**Published:** 2019-07-31

**Authors:** Suk Joon Lee, Yao Chen, Bart Lodder, Bernardo L. Sabatini

**Affiliations:** ^1^Howard Hughes Medical Institute, Department of Neurobiology, Harvard Medical School, Boston, MA, United States; ^2^Department of Neuroscience, Washington University School of Medicine, St. Louis, MO, United States; ^3^Master Program Neuroscience and Cognition, Graduate School of Life Sciences, Utrecht University and Department of Translational Neuroscience, Brain Center, University Medical Center, Utrecht, Netherlands

**Keywords:** FLIM (fluorescence lifetime imaging microscopy), fiber photometry, PKA, dopamine, accumbens

## Abstract

All cells respond to extracellular signals by altering their intracellular biochemical state. In neurons, such signaling regulates many aspects of cell and synapse biology and induces changes that are thought to be important for nervous system development, its adaptation in the face of a changing environment, and ongoing homeostatic maintenance. Although great advances have been made in developing novel fluorescent reporters of intracellular signaling as well as in methods of fluorescence detection for use in freely moving animals, these approaches have generally not been combined. Thus, we know relatively little about how the intracellular biochemical state of neurons, and other cell classes, is dynamically regulated during animals’ behavior. Here we describe a single multi-mode fiber based fluorescence lifetime photometry system (FLiP) designed to monitor the state of fluorescence reporters of biochemical state in freely moving animals. We demonstrate the utility of FLiP by monitoring the lifetime of FLIM-AKAR, a genetically encoded fluorescent reporter of PKA phosphorylation, in populations of direct and indirect pathway striatal projection neurons in mice receiving food rewards. We find that the activity of PKA in each pathway is transiently regulated by reward acquisition, with PKA phosphorylation being enhanced and repressed in direct and indirect pathway neurons, respectively. This study demonstrates the power of FLiP to detect changes in biochemical state induced by naturalistic experiences in behaving animals.

## Introduction

Neurons in the mammalian brain utilize transmembrane signaling to regulate their intracellular state in response to extracellular cues. One prominent mode of such regulation is through the activation of G-protein coupled receptors, which transduce the binding of an extracellular ligand to liberate effector G-proteins and generate second messengers (Greengard, 2001; Kreitzer and Malenka, 2008; Chen et al., 2017). Similarly, Ca-permeable ion channels, such as voltage-gated Ca channels and NMDA-type glutamate receptors, increase intracellular calcium and activate Ca-dependent signaling cascades (Lee et al., 2009; Herring and Nicoll, 2016). Other extracellular signals, including neurotrophins, cytokines, and gasses also rapidly induce changes in cellular biochemical state (Huang and Reichardt, 2001; Regehr et al., 2009; Prieto and Cotman, 2017). Such modulation of intracellular signaling by extracellular signals is central to activity-dependent signaling cascades, including those that mediate synaptic plasticity, dendritic protein translation, the regulation of kinases and phosphatases, and gene-transcription (Nayak et al., 1998; Carvalho et al., 1999; Shaywitz and Greenberg, 1999; Lau et al., 2004; Skeberdis et al., 2006; Lee et al., 2009; Kandel, 2012; Yagishita et al., 2014). Thus, these pathways are thought to be crucial for activity-dependent circuit formation and refinement during development as well as for learning, memory formation, and behavioral adaptation in response to a changing environment (Kandel and Abel, 1995; Davis, 1996; Beninger and Gerdjikov, 2004; Tronson et al., 2006; Doll and Broadie, 2014; Jiang and Nardelli, 2016). To understand how these pathways are activated and how they contribute to these functions, one would ideally monitor the dynamics of the upstream activating second messenger (such as Ca or cAMP), the specific activity of effector enzyme itself (such as the activity state of a kinase), and the state of the enzyme substrates (such as the phosphorylation state of a kinase target).

Protein kinase A (PKA) in the striatum has been proposed to be an essential mediator of dopamine dependent reward-reinforced behaviors in animals (Beninger and Gerdjikov, 2004; Kravitz and Kreitzer, 2012). The dorsal striatum and the ventral striatum nucleus accumbens (NAc) receive dense innervation from dopaminergic neurons located in the substantia nigra pars compacta (SNC) and ventral tegmental area (VTA), respectively (Howe and Dombeck, 2016). Dopamine release in the striatum is generally triggered by positive action outcomes, such as the acquisition of water or food, and, through processes that are still poorly understood, modulates striatal circuits to promote the repetition of the action that led to the good outcome (Schultz et al., 1997; Bromberg-Martin et al., 2010; Schultz, 2013). Central to models of dopamine action on the striatum and reward reinforcement are its differential effects on the two main classes of striatal projection neurons (SPNs) (Berke and Hyman, 2000; Shen et al., 2008; Bromberg-Martin et al., 2010; Kravitz and Kreitzer, 2012). These models propose that dopamine acting through G_α__*s*_-coupled Type 1 dopamine receptors (D1Rs) expressed by direct pathway SPNs (dSPNs) promotes motor action and reward reinforcement by activating PKA in these cells to increase their intrinsic excitability, potentiate synapses, and induce transcription (Gerfen and Surmeier, 2011; Tritsch and Sabatini, 2012). Conversely, dopamine acting through G_α__*i*_-coupled Type 2 dopamine receptors (D2Rs) expressed by indirect pathway SPNs (iSPNs) may induce opposite effects in these cells (Gerfen and Surmeier, 2011; Tritsch and Sabatini, 2012). These push-pull actions of dopamine are thought to induce imbalances in activities and biochemical states of dSPNs and iSPNs that contribute to reward reinforced learning, drug addiction, and Parkinson’s disease (Bromberg-Martin et al., 2010; Kravitz and Kreitzer, 2012). However, due to technical limitations (Goto et al., 2015; Yamaguchi et al., 2015), the dynamic change of PKA activity in dSPNs and iSPNs during behavior is unclear, and thus basic tenets of this model remain untested.

Here, we developed a system to monitor PKA signaling in genetically-defined neurons, such as dSPNs and iSPNs, in freely behaving mice. We exploit a genetically encoded sensor for PKA, FLIM-AKAR (Chen et al., 2014), which contains a GFP-based fluorophore whose lifetime in the excited state changes when the reporter is phosphorylated by PKA. In order to use this sensor in freely moving mice, we designed a single optical fiber system for fluorophore lifetime measurements, building on the previous work that used a pair of optical fibers to monitor the static lifetime of a genetically encoded fluorophore *in vivo* (Cui et al., 2013, 2014). We refer to this approach as Fluorescence Lifetime Photometry (FLiP). We validated this approach by performing bulk measurements of fluorophore lifetime change *in vivo* and demonstrate the ability of FLiP to detect dynamic modulation of PKA activity in the NAc. Our results demonstrate that environmental signals, such as the acquisition and consumption of food, trigger brief and opposing PKA phosphorylation transients in dSPNs and iSPNs. We propose that FLiP can be used with other fluorescence lifetime reporters of biochemical pathways and is a powerful tool for monitoring intracellular biochemical states *in vivo*.

## Results

### Analysis of Fluorescein Standards Using 2P-FLIM and FLiP

A previous study by Cui et al. (2013) measured the fluorescence lifetime of a fluorophore in the brain of a mouse using a dual fiber photometry system in which a single-mode fiber delivered excitation light into the brain and a multi-mode fiber collected light in the brain. The rationale behind this design was that a single-mode fiber provides more stable illumination than a multi-mode fiber, making it more suitable for excitation. Conversely, a multi-mode fiber has higher light collection efficiency than a single-mode fiber, making it more suitable for fluorescence collection. Since it is difficult to launch a free space laser into a single mode fiber, and two fibers are bulky and more damaging when inserted into the brain, we examined if bulk fluorescence lifetime measurements could be made through a single multimode fiber. In addition, we examined if the sensitivity of such a system was sufficient to detect small changes in fluorescence lifetime of a FÖrster resonance energy transfer (FRET)-based reporter of an intracellular biochemical signal in freely moving animals.

**FIGURE 1:**
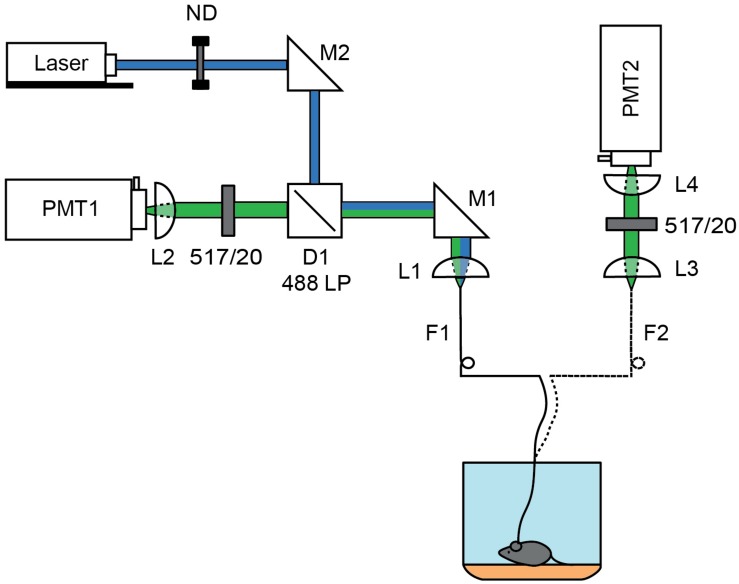
Schematics of 1 and 2 fiber fluorescence lifetime photometry system. A 473 nm pulsed laser (blue) was attenuated using a rotating neutral density filter (ND) and focused into a fiber optic (F1) patch cord using steerable mirror M1 and lens L1. For the 1-fiber based method, fluorescence was collected through the same fiber (F1) using lens L1. Emission light was separated from the excitation light using dichroic D1 (488 LP) and a 517/20 nm emission filter before focusing the fiber face on a photomultiplier tube (PMT1) by L2. The patch cord was connected to the single fiber optic implant inserted into the brain. For the 2-fiber based method, a dual fiber optic implant was used and connected to F1, which was used solely for excitation, and to a second fiber optic (F2) patch cord used to collect fluorescence. The F2 fiber face was imaged onto a PMT (PMT2) using lens L3 and L4 which were separated by a 517/20 nm emission filter. In each case, the PMT was connected to a time correlated single photon counting board.

**FIGURE 2:**
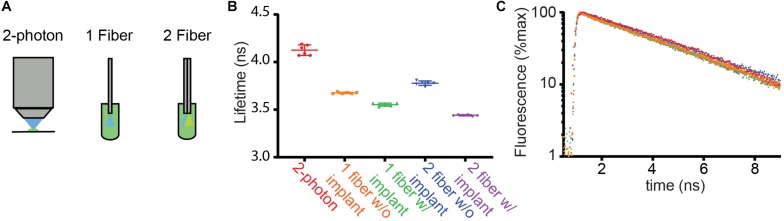
Comparison of fluorescence lifetimes across measurement modalities. **(A)** Schematics of methods used to measure the fluorescence lifetime of fluorescein in solution (4 μg/ml). Lifetime was measured from a droplet under the objective of a 2 photon fluorescence lifetime imaging microscope (2-photon) using pulsed 920 nm excitation. In addition, the fiber optic based setups described in Figure 1 were used to measure lifetime with the fiber ends dipped into the fluorescein solution. **(B)** Average fluorescence lifetimes measured with the arrangements schematized in **(A)**. The 1- and 2-fiber measurements were made with and without the patch cord connected to the corresponding implants that are normally inserted into the brain. The results of each of 6 measurements (dots) as well as the averages and SDs are shown (lines). **(C)** Example fluorescence lifetime histograms for each recording color-coded as in **(B)**.

To test these possibilities, we built a FLiP system that relays pulsed excitation light to an optical fiber and detects fluorescence emission by time correlated single photon counting (Figure 1). The FLiP setup was versatile and could be used with either a pair of multi-mode optical fibers for independent excitation and emission paths or with a single multi-mode optical fiber for both excitation and emission light. We used the setup to measure the lifetime of fluorescein in a standard solution and compared the measurements to those made by a conventional 2-photon fluorescence lifetime imaging microscope (2P-FLIM) (Figure 2A). On average, the lifetime of a fluorescein standard was underestimated by FLiP (3.4–3.8 ns) compared to with 2P-FLIM (4.1 ns) (Figures 2B,C). This deviation likely arises from the contribution of fiber autofluorescence, which has a shorter lifetime [2.7 ± 0.1 ns (SD), *n* = 6, measured by launching laser through the fiber into an optical dump] than fluorescein. It is also possible that because 2P-FLIM excitation light is focused onto a very small volume of fluorescein solution, it may quickly bleach fluorophore molecules and effectively prevent homo-FRET. Since the fiber-based methods distribute excitation light to a large volume of fluorescein solution, they may suffer more from autoquenching.

We measured lifetimes with FLiP with optical fibers directly inserted into the fluorescein solution or with an implant that would normally be placed in the brain (Figures 2B,C, “w/implant” vs. “w/o implant”). For both the single and dual fiber systems, the addition of the implant lowered the lifetime further, likely reflecting additional autofluorescence of the fiber and the glue in the fiber optic implant.

These measurements revealed that the single fiber setup with a fiber implant reported a fluorescein lifetime measurement closer to the 2P FLIM value than the double fiber setup with a fiber implant, suggesting that the double fiber system does not provide a significant advantage over a single fiber system. Overall, fluorescein lifetime measurements demonstrate that a single multi-mode fiber-based lifetime measurement can measure a lifetime close to a true value in a stable manner [3.55 ± 0.02 ns (SD) of fiber measurements vs. 4.13 ± 0.06 ns of 2P FLIM measurements, *n* = 6]. Nevertheless, measuring the absolute lifetime of the targeted fluorophore may be more challenging with FLiP than with 2P-FLIM because of the contribution of fiber autofluorescence as well as the larger contribution of tissue autofluorescence to FLiP measurements (compared to that from a small excitation volume in 2P-FLIM). However, this is not a limitation for many studies in which changes in fluorescence lifetime will be used to measure dynamic engagements of a biochemical pathway *in vivo*.

### FLiP Can Detect Dopamine Receptor Driven Activation of dSPN PKA Activity *in vivo*

To test if single fiber FLiP can detect a biologically meaningful signal, we used it to measure the lifetime of a PKA activity reporter (FLIM-AKAR) that we previously developed (Chen et al., 2014). Briefly, FLIM-AKAR is a FRET-based PKA activity reporter consisting of a donor fluorophore and a dark acceptor. When the conformation of the sensor changes due to its phosphorylation by PKA, it brings the donor and the acceptor closer together, enhancing intramolecular FRET and reducing the fluorescence lifetime of the donor. We have shown that, when monitored by 2P-FLIM, FLIM-AKAR fluorescence lifetime decreases upon PKA activation and increases upon PKA inactivation in neurons in brain slices (Chen et al., 2014).

**FIGURE 3:**
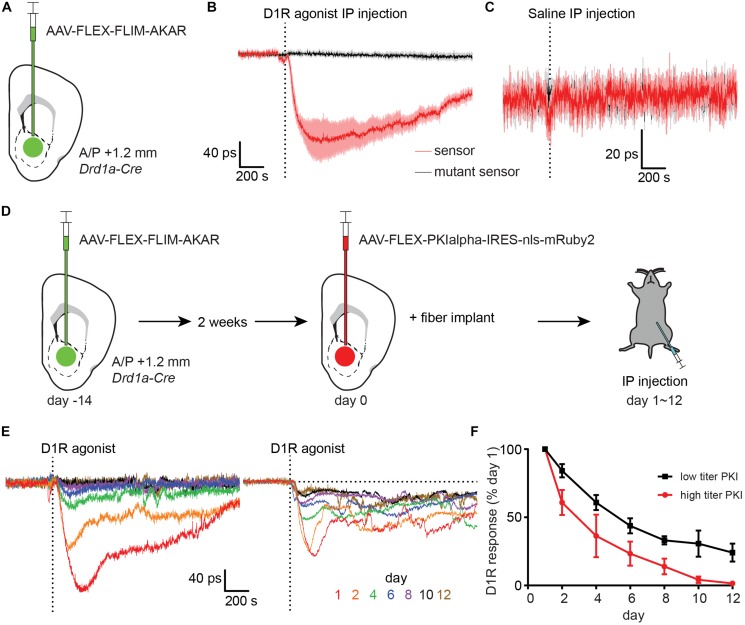
Type 1 dopamine receptor agonists increase PKA phosphorylation in NAc dSPNs. **(A)** Schematic of a coronal section at 1.2 mm depicting viral injection of AAV-FLEX-FLIM-AKAR into the NAc of a *Drd1a-Cre* mouse. An optical fiber was implanted 200 μm above the injection site. **(B)** Changes in fluorescence lifetime of FLIM-AKAR expressed in dSPNs of a *Drd1a-Cre* mouse that was intraperitoneally (IP) injected with a Type 1 dopamine receptor (D1R) agonist. The changes over time in fluorescence lifetime measured in 1-s interval relative to the baseline (pre-injection) period are plotted. The time when the injection finished is indicated by the dashed vertical line. For FLIM-AKAR (red), a decrease in lifetime represents an increase in phosphorylation by PKA. A similar measurement was made using FLIM-AKAR^*T*391*A*^ which has a point mutation at the PKA phosphorylation site (black). For the sensor and mutant sensor, the average responses (line) and SEM (shaded regions) across seven mice are shown. **(C)** As in **(B)**, the responses of FLIM-AKAR (red) and the phosphorylation site mutant FLIM-AKAR^*T*391*A*^ (black) to IP injection of saline. For each, data collected across four mice are shown. **(D)** Experimental design for using virally-transduced PKI to validate the specificity of the FLIM-AKAR lifetime changes in dSPNs following D1R agonist injection. A *Drd1a-Cre* mouse was first injected with AAV-FLEX-FLIM-AKAR to express the reporter in dSPNs (left). After 2 weeks had elapsed to achieve strong expression of the reporter, the same mouse and region were injected with AAV-FLEX-PKIalpha-IRES-nls- mRuby2 (middle). An optical fiber was implanted during the same surgery used for the second viral injection. The responses to IP D1R agonist injection were measured (right) over the subsequent 1–12 days. **(E)** D1R agonist induced changes in FLIM-AKAR lifetime across multiple days from example mice injected with high (left) or low (1/10^th^ of high, right) titer PKI virus. **(F)** The amplitudes of D1R agonist induced changes in FLIM-AKAR lifetime relative to that on day 1 across multiple days for high (red, *n* = 3 mice) and low (black, *n* = 4 mice) titer PKI virus groups. Error bar = SEM.

We expressed FLIM-AKAR in dSPNs in the NAc by injecting an adeno-associated virus (AAV) that expressed the sensor in a Cre-dependent manner (AAV-FLEX-FLIM-AKAR) into *Drd1a-Cre* mice and implanted a single optical fiber into the NAc (Figure 3A). After 2 weeks of expression of the reporter virus, we injected a Type 1 dopamine receptor (D1R) agonist (SKF 81297 hydrobromide, 10 mg/kg IP) and measured the lifetime change in the fluorescence signal collected from the NAc in freely moving animals. The fluorescence lifetime of FLIM-AKAR showed a significant reduction (∼0.15 ns) suggesting an increase in PKA activity in dSPNs after D1R agonist injection (Figure 3B). The magnitude of the lifetime change was comparable to that observed previously in brain slices with activation of PKA (Chen et al., 2014).

The D1R-agonist induced fluorescence lifetime change did not occur in mice expressing a mutant reporter (FLIM-AKAR^*T*391*A*^) in dSPNs, which has a point mutation at its consensus PKA phosphorylation site (Figure 3B). This demonstrates that the phosphorylation of the sensor at this site is required for the observed lifetime change. Furthermore, IP injection of saline did not produce a comparable transient, suggesting that the lifetime change in D1R agonist experiments is due to the effect of the drug rather than a general stress caused by scruffing and injection (Figure 3C). Interestingly, there was a small but significant change in FLIM-AKAR lifetime at the time of D1R agonist or saline IP injection that was much smaller and shorter-lived than the long-lived D1R agonist response. This small signal was not seen in FLIM-AKAR^*T*391*A*^ expressing mice suggesting that there is a general effect of IP injection that increases dSPN PKA activity.

To further validate the specificity of the reporter fluorescence lifetime change *in vivo*, we employed PKI, which is one of the most specific inhibitors of PKA currently available (Dalton and Dewey, 2006; June, 2012). We had previously developed an AAV that expresses PKI in a Cre-dependent manner (Chen et al., 2014), allowing specific antagonism of PKA in a genetically-defined population. We first injected AAV-FLEX-FLIM-AKAR into the NAc of a *Drd1a-Cre* mouse and waited 2 weeks to achieve stable expression of the reporter (Figure 3D). We subsequently injected AAV-FLEX-PKIalpha-IRES-nls-mRuby2 into the same location and performed fiber implantation. This approach allowed us to measure PKA dynamics in dSPNs as PKI expression increased starting the day after fiber implantation (day 1). During days 1–12, we have repeated IP delivery of D1R agonist and observed a gradual decrease in the magnitude of the evoked signal over time. With a high PKI virus titer cohort, the D1R agonist response was eliminated by day 10 (Figures 3E,F). With a 10-fold lower PKI virus titer cohort, the D1R agonist response was reduced to 30% of its amplitude on day 1 (Figures 3E,F). This reduction in FLIM-AKAR response is likely caused by the intended PKI effect of PKA inhibition as *post hoc* histological analysis showed minimal signs of neuronal death and confirmed expression of FLIM-AKAR.

Thus, given its dependence on PKA phosphorylation site and its blockade by PKI expression, we conclude that D1R-agonist induced decrease in FLIM-AKAR lifetime reflects bona fide activation of PKA in dSPNs. Furthermore, these results confirm the ability of FLiP to report dynamic modulation of endogenous biochemical signaling cascades in genetically-defined neurons in freely moving animals.

### FLiP Can Detect Modulation of PKA Activity in dSPNs and iSPNs Induced by a Natural Stimulus

**FIGURE 4:**
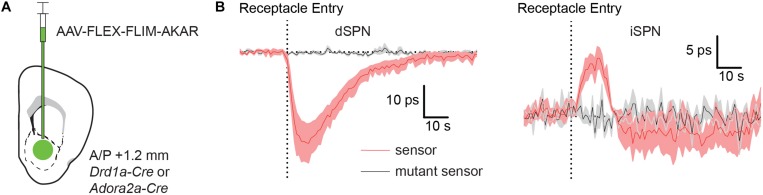
Food rewards rapidly increase and decrease PKA phosphorylation in dSPNs and iSPNs, respectively. **(A)** Schematic of a coronal section at 1.2 mm depicting viral injection of AAV-FLEX-FLIM-AKAR into the NAc of a *Drd1a-Cre* or *Adora2a-Cre* mice. An optical fiber was implanted 200 μm above the injection site. **(B)** Fluorescence lifetime changes of FLIM-AKAR (red) expressed in a dSPNs in *Drd1-Cre* mice (left) or iSPNs in *Adora2a-Cre* mice (right) when the animals were given a food reward (dashed vertical line = time point of food pellet receptacle entry). The mice were food restricted at least a day before the food reward response was measured. The patterns show an increase in PKA phosphorylation in dSPNs (*n* = 4 mice) and a decrease in iSPNs (*n* = 5 mice). No change is seen in mice expressing FLIM-AKAR^*T*391*A*^ (black) in either dSPNs (*n* = 3 mice) or iSPNs (*n* = 4 mice). Shaded area = SEM of averages across mice. For each mouse, data were collected across 10 trials.

Although the D1R agonist experiment is a good proof-of-principle test for FLiP, the result of this experiment does not guarantee that the system will be able to pick up a change in PKA activity caused by a natural stimulus, which is likely to be smaller than the long-lasting effect of D1R agonism. Natural stimuli may induce signals that are more similar in kinetics and amplitude to the signals seen following IP saline injection (Figure 3C). Therefore, we tested if FLiP can detect changes in PKA activity in dSPNs and iSPNs in mice that receive a food reward. To compare PKA dynamics in dSPNSs and iSPNs, we expressed FLIM-AKAR in each population using *Drd1a-Cre* and *Adora2a-Cre* mice, respectively (Figure 4A). Two-three weeks after virus injection and fiber implantation, mice were food restricted for at least 1 day before the food reward response was measured. We subsequently measured the change in the fluorescence lifetime of FLIM-AKAR in these mice when we gave them a small food pellet (20 mg) from a food receptacle in an otherwise empty chamber. Access to food in these mice was expected to elicit a dopamine transient in the NAc, resulting in subsequent activation G_α__*s*_-coupled dopamine receptors on dSPNs and G_α__*i*_-coupled dopamine receptors on iSPNs.

Indeed, FLIM-AKAR lifetime measured in *Drd1a-Cre* mice significantly decreased at the time of food consumption, as expected from an increase in dSPN PKA activity (Figure 4B). Conversely, FLIM-AKAR lifetime in *Adora2a-Cre* mice increased at the time of food consumption, marking a reduction in PKA activity in iSPNs (Figure 4B). Neither signal was seen in mice expressing FLIM-AKAR^*T*391*A*^, confirming that the lifetime changes observed in FLIM-AKAR expressing animals on food consumption did not come from movement artifacts or changes in tissue autofluorescence.

The opposite changes in PKA activity in dSPNs and iSPNs are consistent with expected phasic dopamine release at the time of unexpected reward delivery leading to activation and suppression of PKA in each neuron class, respectively. Furthermore, these results demonstrate the utility of FLiP in monitoring changes in PKA activity *in situ* triggered by physiological stimuli in behaving animals.

### Expression of FLIM-AKAR Minimally Perturbs PKA Activity Dynamics

A concern with reporters of physiological processes is that their expression may perturb the process of interest. For example, expression of a phosphorylation substrate could slow phosphorylation or dephosphorylation if kinases or phosphatases are in rate-limiting quantities. Such effects are typically seen as slowing of the signal as the levels of the reporters are increased. To examine if the expression of FLIM-AKAR perturbs the dynamics of PKA-dependent phosphorylation, we performed multi-day measurements of FLIM-AKAR lifetime changes in response to a food reward as the level of the reporter increased.

**FIGURE 5:**
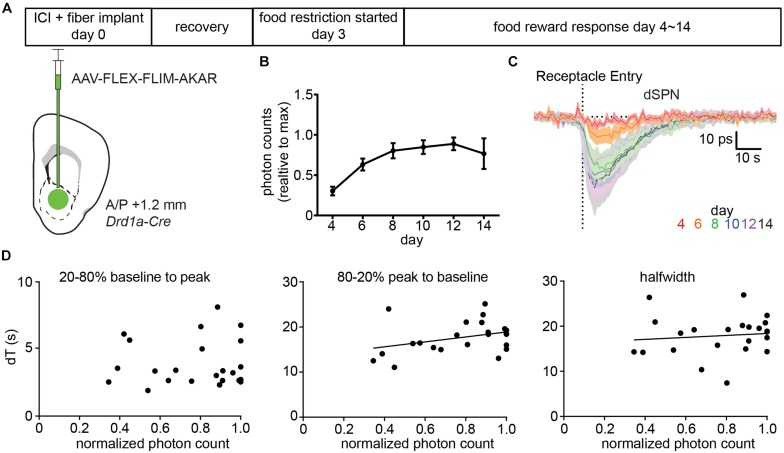
Expression of FLIM-AKAR minimally perturbs PKA activity dynamics. **(A)** Schematic of the experimental design for testing the effects of FLIM-AKAR expression levels on response kinetics. First, a *Drd1a-Cre* mouse was injected with AAV-FLEX-FLIM-AKAR intracranially and was implanted with a fiber. After 3 days of recovery, the mouse was started on food restriction. Starting on day 4 after surgery (1 day after the beginning of food restriction), food induced changes in lifetime of FLIM-AKAR expressed in dSPNs were measured for 10 days. After 10 days, the fluorescence intensity reached its maximum for all mice, suggesting viral sensor expression had reached its maximum **(B)**. **(B)** Average photon counts of FLIM-AKAR fluorescence under the fixed excitation power across multiple days, normalized for each mouse by its maximum photon count. Error bar = SEM across *n* = 5 mice. **(C)**. Average food-induced change in lifetime of FLIM-AKAR expressed in dSPNs of *Drd1a* -Cre mice across days. The dashed vertical line indicates the time when the animals entered the food pellet receptacle. Shaded area = SEM across *n* = 5 mice. For each mouse, data were collected across 6–10 trials/day. **(D)** For each mouse on each day, the FLIM-AKAR response kinetics were quantified by measuring the time required for the signal to reach 80% of the maximum valley amplitude from 20% of the maximum valley amplitude (20–80% baseline to peak) (left) and the time to return to 20% of the maximum valley amplitude from 80% of the maximum valley amplitude (80–20% peak to baseline) (middle). In addition, the halfwidth (right) of the FLIM-AKAR lifetime response was measured. Each parameter is plotted as a function of the baseline fluorescence photon counts measured in each mouse for each day during periods when the animal was not eating. *N* = 5 mice with 6–10 trials acquired for each data point. Measurements were analyzed when the fluorescence intensity reached 30% of its maximum as they were too noisy at lower intensities.

We injected AAV-FLEX-FLIM-AKAR into *Drd1a-Cre* mice (Figure 5A) and, after 3 days of surgery, started food restriction on the mice. Starting on the day after the beginning of food restriction, we measured FLIM-AKAR lifetime changes in response to a food reward across multiple days as the expression of FLIM-AKAR increased. The increase in FLIM-AKAR expression was estimated from the basal level of fluorescence under constant excitation power (Figure 5B), which underestimates the true fold change in the reporter expression as it ignores that some fraction of the fluorescence arises from autofluorescence of the fiber and tissue. The daily average lifetime plot (averaged from five mice) showed no significant slowing of the dSPN PKA activity dynamics reported by FLIM-AKAR (Figures 5C,D), even as the amplitude of the lifetime change increased over an ∼3-fold. Furthermore, from day to day, there was no strong positive correlation between normalized photon counts and the time to maximal deviation from baseline (20–80% baseline to peak, *R* ^2^ = 0.00078), the time to return to baseline (80–20% peak to baseline, *R* ^2^ = 0.12), or the half-width of the signal (*R* ^2^ = 0.012) (Figure 5D). Overall, we find no sign of limiting of PKA or phosphatase activity caused by the sensor expression. Of note, the fact that the fluorescence lifetime response increases between day 4∼day10 and stabilizes after day 10 suggests that an accurate and stable measurement of fluorescence lifetime change requires a certain level of fluorophore concentration, which ensures the majority of the detected photons come from the sensor rather than the background (fiber and tissue autofluorescence).

## Discussion

Although many reporters of intracellular second messengers and their downstream signaling cascades have been developed, relatively little is known about the dynamic change of intracellular biochemical signaling other than calcium during behavior. Fluorescence lifetime imaging microscopy (FLIM), a FRET-based imaging approach, has been extensively used to study intracellular signaling in neurons *ex vivo* (Yasuda et al., 2006; Lee et al., 2009; Laviv et al., 2016; Chen et al., 2017; Tang and Yasuda, 2017). FLIM relies on detecting inter- or intra-molecular interactions between a donor and acceptor fluorophore through the FRET dependent change in the fluorescence lifetime of the donor. Genetically-encoded FLIM-compatible reporters have been developed for many kinases and second messengers (Yasuda et al., 2006; Lee et al., 2009; Klarenbeek et al., 2011; Chen et al., 2014; Laviv et al., 2016; Tang and Yasuda, 2017; Colgan et al., 2018; Ma et al., 2018). Inspired by these studies, we developed a single multimode fiber-based method to monitor intracellular signaling in freely-moving mice. We used fluorescence lifetime photometry (FLiP) to examine the dynamics of protein kinase A (PKA) signaling in specific population of neurons in the NAc. The PKA activity reporter that we used, FLIM-AKAR, has been heavily optimized for fluorescence lifetime measurements in the previous study (Chen et al., 2014). We demonstrate that FLiP, using a multimode fiber for both the fluorescence excitation and emission path, provides stable and reproducible measurements of the fluorescence lifetime of FLIM-AKAR. Furthermore, we were able to reveal modulation of the PKA phosphorylation state following pharmacological and behavioral stimuli, confirming the sensitivity of the approach to detect dynamics of intracellular biochemical signaling in behaving mice.

### Single Fiber FLiP

The comparison of the lifetimes of a fluorescein standard measured by FLiP to those measured by 2P-FLIM shows that fiber photometry systematically underestimates lifetimes. We suggest that this is likely due to autofluorescence present in the fiber optic, which typically has a shorter lifetime (∼2.7 ns) than fluorescein (∼4.1 ns). This conclusion is supported by the finding that adding the brain fiber implant to the end of the patch cord lowers the measured lifetime further, likely reflecting the additional fluorescence derived from this device. Nevertheless, utilizing a single multimode fiber for excitation light delivery to the brain and collection of fluorescence from the brain, we were able to measure a biologically meaningful fluorescence lifetime change.

Our results demonstrate that single-fiber FLiP is able to detect pharmacologically- and behaviorally-induced changes in PKA-dependent phosphorylation in genetically-defined populations of neurons in the NAc. Based on the results presented above, we propose that the relatively simple single-fiber system presented here provides a powerful method for the analysis of intracellular signaling cascades in behaving animals.

### Comparison to Other Approaches

Real-time reporters of biochemical processes typically rely on intra- or inter-molecular conformational changes induced by an enzymatic modification, such as phosphorylation, or binding of a bioactive molecule. Some reporters, such as GCaMP, alter their brightness on activation due to a change in absorption cross-section or quantum yield, which typically does not alter the fluorescence lifetime of the fluorophore. These reporters are usually monitored using a single excitation and emission wavelength. When these reporters are used with fiber photometry to collect bulk signals in moving animals, care is needed to ensure that fluorescence dynamics reflect the target biological processes, as opposed to fluorescence changes induced by movement of the optical fiber, changes in pH in the tissue, or hemodynamic alterations that affect photon absorption and scattering (Hires et al., 2008; Cui et al., 2013; Valley et al., 2019).

On the other hand, with two-color FRET sensors, the fluorescence intensities of the acceptor and the donor are both monitored, and their ratio is calculated to deduce changes in biochemical states. Such approach is difficult to implement *in vivo*, as many factors, such as differential bleaching of each fluorophore, overlap in the emission or excitation spectra, differential sensitivity of each spectrum to hemodynamic changes, and differential contribution of autofluorescence to each emission spectrum, will alter the ratio of donor and acceptor fluorescence intensity.

For FRET-based FLIM reporters, activation of the biochemical process alters the fluorescence lifetime of the donor fluorophore, which allows their monitoring by FLiP and other lifetime detection methods. Single fluorophore FLIM method avoids many of the complications of two-color FRET method mentioned above as the FLIM measurement – fluorescence lifetime of the donor – is in principal independent of the concentration of the donor molecules, the excitation efficiency that depends on change in illumination, and the efficiency with which the fluorescence is collected. In practice, this holds true as long as the contribution of background fluorescence to the detected signal is minimal compared to that of the sensor (the majority of the detected photons come from the donor fluorophore).

Fluorescence lifetime imaging microscope and, by extension, FLiP are not impervious to artifacts, and controls for the specificity of observed lifetime transients need to be established as they were done here using a variety of controls. Of particular concern is the non-negligible contribution of autofluorescence from the optical fiber, which can affect the measured fluorescence lifetime if the reporter is dim or rapidly bleaching, compared to the autofluorescence. In our experience, this concern can be avoided by using low excitation light levels that minimize reporter bleaching. Nevertheless, because of the contribution of autofluorescence, we avoid translating absolute lifetime measurements into ratios of active and inactive reporter, as it is often done with low-background imaging modalities such as 2P-FLIM.

### PKA Dynamics in the NAc

We find that pharmacological activation of Type 1 dopamine receptors (D1Rs) using peripheral administration of an agonist induces robust and long-lived (>20 min) increases in PKA dependent phosphorylation in D1R-expressing dSPNs of the NAc. This is consistent with the GPCR biology of D1Rs: they couple to Gαs to increase cAMP concentration and thus activate PKA. The dependence of the reporter response on the PKA phosphorylation site of the reporter and its blockade by expression of PKI, demonstrate that the reporter response results from PKA phosphorylation of FLIM-AKAR. As D1Rs couple through G_α__*s*_ to increase cAMP concentration and thus activate PKA, this was the expected result. In addition to confirming the sensitivity of fiber-based measurements of fluorescence lifetime *in vivo*, these data reveal the *in vivo* pharmacokinetics of SKF 81297 hydrobromide, which is widely used in behavioral experiments. Because FLIM-AKAR can report orders-of-magnitude briefer (∼20 s) behaviorally triggered PKA transients (see below), the kinetics of PKA activation reported by the sensor following D1R agonism likely reflect the duration of exposure of NAc dSPNs to the drug. We propose that analysis of biochemical state using *in vivo* FLiP can be an important tool to determine the brain penetrance, exposure time, or mechanism of action of drugs targeting the central nervous system.

Delivery of a reward, in the form of a food pellet to food-restricted animals, transiently increases and decreases PKA activity in dSPNs and iSPNs in the NAc, respectively. These opposite changes are predicted based on the expression of G_α__*s*_- and G_α__*i*_-coupled receptors in dSPNs and iSPNs, respectively. As the kinetics of these signals are not artificially prolonged by the level of reporter expression, they provide an estimate of the kinetics of combined PKA/phosphatase action on diffusible PKA substrates in the cell. Thus, food reward induced changes in PKA dependent phosphorylation in dSPNs and iSPNs, for ∼40 and ∼20 s, respectively, which provides an upper estimate of the kinetics of the intracellular biochemical change. In addition, we find that rewards lower phosphorylation state in iSPNs, confirming that, in these cells, there is a basal phosphorylation of PKA substrates, which can be reversed by inhibition of adenylyl cyclases via G_α__*i*_-coupled GPCRs such as Type 2 dopamine receptors.

### Summary and Outlook

The fluorescence lifetime photometry system described here is relatively simple to implement. As it is based on a single multi-mode fiber, it is compatible with low cost launches that couple the laser into the fiber. Future improvements may include replacing the expensive time correlated single photon counting boards with more affordable high-speed field-programmable gate arrays (FPGAs) that are more flexible and do not suffer from dead time, which can increase sampling rates. The system presented here can detect behaviorally and pharmacologically induced transients in intracellular biochemical state in genetically-defined neurons. Thus, it will permit further studies of the intracellular biochemical mechanisms of many brain processes, such as those linking experience to cellular plasticity, mediating activity dependent circuit regulation, and by which hormonal and metabolic signals influence cellular states.

## Materials and Methods

Experimental manipulations were performed in accordance with protocols approved by the Harvard Standing Committee on Animal Care following guidelines described in the US National Institutes of Health Guide for the Care and Use of Laboratory Animals. Inhaled isoflurane was used as anesthesia.

### 2-Photon Fluorescence Lifetime Imaging Microscopy (2P-FLIM)

2-Photon fluorescence lifetime imaging microscopy (Figure 2) was carried out using a custom microscope as previously described (Chen et al., 2014). 920 nm excitation light (Chameleon Vision II, 80MHz, Coherent, Santa Clara, CA, United States) was used to 2-photon excite fluorescein dissolved in water, and resulted fluorescence was collected in the 500–550 nm bank using a 525/25 nm emission filter (Semrock) and a PMT (H7422-40 MOD Hamamatsu).

### Fluorescence Lifetime Photometry (FLiP)

Fluorescence lifetime photometry was carried out as illustrated in Figure 1. All filters in the system were made by Semrock. The 473 nm laser BDS-473-SM-FBE [Becker and Hickl (BH)] operated at 50 Mhz was used as a pulsed light source. For detection, a high speed hybrid PMT, HPM-100-07-Cooled (BH) controlled by DCC-100-PCI (BH) was used. The PMT was connected to SPC-830 (BH), a time correlated single photon counting (TCSPC) board, which detects the time delay between the pulsed excitation and the photon detection by the PMT. The data was collected by a custom software in MATLAB, which calculated the average lifetime of detected photons at 1 s intervals. This interval for average lifetime measurements was empirically determined to have enough photons to accurately estimate the lifetime (>200,000 photons/measurement) without running into a photon count limit (<1,000,000 photons/s) of the TCSPC board. The typical excitation power needed to generate an appropriate amount of photons for TCSPC was 0.6∼1 μW (measured at the output end of the patch cord).

For the estimation of the absolute lifetime of a fluorescein solution standard (Figure 2), we fit a single exponential decay to the lifetime histogram. For the data presented in Figure 2, we used the time constant of this single exponential decay as the measure of lifetime. For the estimation of the change in lifetime of the sensor *in vivo* (Figures 3–5), we calculated, for each 1 s time bin, an average lifetime by measuring the mean photon arrival time (the population mean of the delay between the pulsed excitation and the fluorescence photon arrival as described in the equation below), as discussed in the previous study by Lee et al. (2009).

$$\tau_{m}|=<t>-t_{0}=\frac{\int 𝑑t\cdot t\,F\,\left(t\right)}{d\,t\cdot F\,\left(t\right)}-t_{0}$$

Here, F(t) is the photon count of a fluorescence lifetime decay curve at time bin t, and t_0_ is the offset of the lifetime histogram, which can be estimated by fitting a double exponential curve to a lifetime histogram once in the beginning of the experiment.

For this calculation, we chose a time range (0–8 ns) in a lifetime histogram that was minimally contaminated by a secondary fluorescence peak created by the autofluorescence of a fiber. The length of the patch cord was chosen to maximize the time separation between the sensor fluorescence peak and the fiber autofluorescence peak (∼10 ns time delay for light to travel from the one end of the patch cord to the other end and back).

The lifetime of each fluorophore was reported as a change in lifetime (delta lifetime), which was calculated by subtracting the average lifetime of a baseline period (a period in graphs before the event of interest) from the average lifetime transient.

### Surgery

*Drd1a-Cre* or *Adora2a-Cre* mice with C57BL/6J background from MMRRC UC Davis were used for all *in vivo* experiments. AAV1-FLEX-FLIM-AKAR (2.14 × 10^13^ gc/ml, 300 nl), AAV1-FLEX-FLIM- FLIM-AKAR^*T*391*A*^ (7.67 × 10^12^ gc/ml, 300 nl), AAV-FLEX-PKIalpha-IRES-nls-mRuby2 (3.72 × 10^13^ gc/ml for low titer and 3.72 × 10^14^ gc/ml for high titer, 600 nl) were stereotactically injected at +1.2 mm (A/P), +1.3 mm (M/L) from bregma at a depth of 4.1 mm from brain surface. A fiber optic implant (MFC_200/230-0.37_4.5mm_MF1.25_FLT mono fiber optic cannula or DFC_200/245-0.37_5.5mm_DF0.7(2.5)_FLT dual fiber optic cannula from Doric Lenses) was implanted at the same coordinate at a depth of 3.9 mm from the brain surface. The dual fiber optic implant was custom made to place the two fibers adjacent to each other without any gap and thus to maximize the overlap between the excitation and collection volumes.

### Pharmacology

SKF 81297 hydrobromide (10 mg/kg mixed in 0.1 ml of saline/10 g of mouse) was injected intraperitoneally (IP) to activate Type 1 dopamine receptors (D1R) (Figure 3). Saline (0.1 ml of saline/10 g of mouse) was injected IP for the controls trials.

### Behavior

A mouse connected to a patch cord was allowed to freely move in an 8 × 16 inch behavior box, which contained a pellet receptacle in one wall. Receptacle entry was detected by an infrared sensor installed inside the receptacle. The time of beam break was used to align the fluorescence lifetime measurements across trials and to analyze changes in lifetime caused by food rewards.

## Data Availability

The datasets generated for this study are available on request to the corresponding author.

## Ethics Statement

The animal study was reviewed and approved by Harvard Medical School IACUC.

## Author Contributions

BS conceived, analyzed, and wrote the manuscript. SL collected the data, conceived, analyzed, and wrote the manuscript. YC conceived and edited the manuscript. BL collected the data and analyzed the manuscript.

## Conflict of Interest Statement

The authors declare that the research was conducted in the absence of any commercial or financial relationships that could be construed as a potential conflict of interest.
